# Differential effects of Tra2β isoforms on HIV-1 RNA processing and expression

**DOI:** 10.1186/1742-4690-10-S1-P17

**Published:** 2013-09-19

**Authors:** Craig Platt, Maria Calimano, Josip Nemet, Jodi Bubenik, Alan Cochrane

**Affiliations:** 1 Molecular Genetics, University of Toronto, Toronto, Ontario, Canada

## Background

Balanced processing of HIV-1 RNA is critical to virus replication and is regulated by host factors such as SR proteins. To examine the role of the SR-related proteins Tra2a and Tra2β in regulating viral gene expression, we used both overexpression and depletion analysis to examine how these factors impacted HIV-1 RNA splicing, transport and expression and used mutation to identify the protein domains involved.

## Materials and methods

HEK 293/293T cells were transfected with plasmids expressing HIV-1 provirus and with vectors expressing Tra2a, Tra2β, or mutants thereof. Effects on viral gene expression and RNA processing were monitored by western/northern blot, *in situ* hybridization, and RT-PCR. To examine effects of Tra2β depletion, cells were transduced with lentivirus expressing control or anti-Tra2β shRNAs and similar analyses used to monitor changes in HIV-1 RNA processing and expression.

## Results

Overexpression of either Tra2a or Tra2β results in a marked reduction in HIV-1 Gag/Env expression, an effect associated with changes in HIV-1 RNA accumulation and a block to export of HIV-1 genomic RNA. Mutagenesis to define the domains critical for the inhibitory activity revealed that a natural isoform of Tra2β (Tra2β3), lacking the N-terminal RS domain, also suppressed HIV-1 expression but had very different effects on viral RNA processing at both the level of accumulation of the various viral RNAs (unspliced, singly spliced and multiply spliced) as well as splice site usage. In contrast, variants lacking the C-terminal RS domain or point mutants that disrupt RNA binding had no effect. Tests to define elements that mediate the response determined that the ESE3 and ESS$ within the terminal exon of HIV-1 are not required for the effects observed. The functional differences between the Tra2β isoforms were also observed in the context of another RNA substrate indicating that these factors have distinct functions within the cell. Finally, we demonstrate that Tra2β depletion results in a selective reduction in HIV-1 Env expression that is correlated with decreased accumulation of the corresponding viral RNA.

## Conclusions

Together, these findings indicate that Tra2a/β can play important roles in regulating HIV-1 RNA metabolism and expression, suggesting that modulation of its activity could be used to suppress virus replication. Furthermore, we demonstrate that the different isoforms of Tra2β (β1 and β3), while inducing similar reductions in HIV-1 Gag and Env expression appear to achieve this end by modulating viral RNA processing in different ways.

